# Paramedic Education and Training for the Management of Patients Presenting with Low-Acuity Clinical Conditions: A Scoping Review

**DOI:** 10.3390/healthcare12020176

**Published:** 2024-01-11

**Authors:** Anthony Carnicelli, Anne-Marie M. Williams, Dale G. Edwards

**Affiliations:** 1 Tasmanian School of Medicine, College of Health and Medicine, University of Tasmania, Hobart, TAS 7000, Australia; annemarie.williams@utas.edu.au (A.-M.M.W.); dale.edwards@utas.edu.au (D.G.E.); 2 Clinical Services, Ambulance Tasmania, Cambridge, TAS 7170, Australia

**Keywords:** paramedic, education, training, low acuity, alternative pathways

## Abstract

Ambulance services around the world are increasingly attending to calls for non-emergency conditions. These lower-acuity conditions do not always require patients to be transported to the emergency department. Consequently, over the past two decades, ambulance services have implemented strategies to support paramedics in diverting non-urgent patients to alternative care pathways. However, assessing and managing low-acuity conditions can be challenging for paramedics, especially when education and training has traditionally focussed on emergency care. This scoping review explores the education and training provided to paramedics on low-acuity clinical conditions and the use of alternative care pathways. The Preferred Reporting Items for Systematic Reviews and Meta-Analyses extension for Scoping Reviews was applied. The databases searched included Scopus, CINAHL, Embase, Emcare, and MEDLINE (PubMed). The search identified one-hundred sixty-six records, with a total of nine articles reviewed after the removal of duplicates and the screening process. The articles were diverse, with education and training ranging from university degrees for extended care practitioners to short in-service-based training for a suite of protocols or assessment tools. However, the literature addressing education and training on low-acuity conditions and alternative care pathways is limited, with the type and length of education programs appearing to influence practice. There is a need for further research to establish a low acuity education model.

## 1. Introduction

Traditionally, paramedics respond to calls in an ambulance for life-threatening illnesses or injuries [1]. However, over the past two decades, this has rapidly changed with an increasing proportion of patients requesting assistance for low-acuity clinical conditions [1,2]. Now, in addition to providing basic and advanced life support, paramedics in many countries have become “mobile healthcare professionals”, who are required to assess and manage a range of chronic, social, non-urgent, and mental healthcare needs [1,3]. The role of a paramedic is unique and complex; working in uncontrolled and unpredictable environments, they are required to rapidly gather, interpret, and continuously re-evaluate both patient and scene information, as well as formulate treatment and transport decisions [4,5]. Additionally, various aspects of logistics and resource management is also factored into their decision-making processes [6]. A paramedic must constantly analyse whether they are making appropriate and safe decisions for their patients, often with limited equipment and resources [4,5]. In acute or time-critical situations, these decisions, particularly the need to transport a patient to the emergency department (ED), are mostly clear [7]. However, transport decisions for patients presenting with lower-acuity clinical conditions, which are often complicated by multiple comorbidities or social needs, can be more complex, with higher levels of uncertainty and risk [7,8]. In these circumstances, paramedics often decide to transport the patient at hand to a hospital, as this is perceived to be the safer option, often leading to a medically unnecessary transport [7,9,10,11,12]. 

The increasing need for paramedics to manage lower-acuity presentations is common across Australasia and internationally [2,12,13,14,15,16,17]. In Australia, of close to four million patients seen by emergency ambulance services in 2021-22, approximately 15% were not conveyed to hospital [18]. Research suggests that the percentage of patients calling for an ambulance then deemed to be lower acuity varies and can range from 53% up to 85% [14,19,20]. Low-acuity illness and injury types that paramedics attend to include minor wounds and soft tissue injuries, musculoskeletal back pain, gastroenteritis symptoms, primary headache syndromes, mild allergic reactions, mild upper respiratory tract infections, epistaxis, and social issues [15,21,22,23]. It has become commonplace over the past two decades for paramedics to not convey patients to the ED because of the increasing low-acuity caseload.

With increasing non-urgent caseloads and overcrowded EDs, ambulance services have attempted to assist paramedics with non-conveyance assessment and management by implementing various “treat-and-refer” approaches such as guidelines, protocols, triage tools, and flowcharts [23,24,25,26,27,28,29]. The evaluation of these support tools has highlighted several barriers to their use in the field, including education and training [7,21,25,26,30,31]. Insufficient training has been found to be a significant challenge to paramedic utilisation and compliance, with many paramedics reporting they lacked the confidence to use them or that higher levels of clinical reasoning are required [7,10,25,32]. Similar difficulties with decision-making including confidence have been noted in other healthcare professions, especially when they were faced with unfamiliar medical conditions or situations and uncertainty [33,34]. 

To improve paramedics’ use of non-conveyance guidelines and to increase confidence in low-acuity and non-conveyance decision making, researchers have identified a clear need for education and training [7,26,35,36,37,38]. It is the aim of this scoping review to explore the literature and identify potential knowledge gaps related to paramedic education and training in the use of low-acuity care pathways such as guidelines, protocols, or other methods. These pathways enable paramedics to avoid medically unnecessary conveyance to hospital by allowing them to discharge a patient at the scene and/or refer them to another healthcare service. Any identified gaps in the literature will inform further research in this area, including low-acuity education curriculum development, analysis of low-acuity clinical practice guidelines, and the experiences of paramedics assessing and managing lower-acuity clinical conditions.

For the purpose of this review, a ‘paramedic’ is defined as a non-physician, out-of-hospital healthcare professional working in an ambulance service [39]. Globally, the term paramedic is broad, with various titles, scopes of practice, crew mix, and qualification requirements. The definition includes health professionals such as ambulance nurses who operate in a clinical role comparable to paramedics in Australasian and UK services [40,41,42,43].

## 2. Materials and Methods

The primary research question for this review was the following: “what education and training is provided to paramedics to support their use of alternative care pathways when managing low-acuity patients in the community?”. A scoping review was selected as the appropriate methodology for this study, as the aim was to identify published articles related to the research question, map the key concept(s), and provide a broad overview including current knowledge gaps [44]. This review followed the Joanna Briggs Institute’s (JBI) methodology and utilised the Preferred Reporting Items for Systematic Reviews and Meta-Analyses extension for Scoping Reviews (PRISMA-ScR) [45,46]. The PRISMA-ScR checklist is provided in Table S1 [47]. A protocol for this scoping review has been previously published [48].

The Population, Concept, and Context (PCC) framework (Table 1) was used to inform the eligibility criteria and align these with the research question [46].

### 2.1. Eligibility Criteria

#### 2.1.1. Inclusion Criteria

The articles in this scoping review were related to paramedics working in ambulance systems aligned with the Anglo-American EMS model [49,50]. This operational ambulance model is present around the world, including in Australia, New Zealand, the United Kingdom (UK), United States of America (USA), Canada, Republic of Ireland, South Africa, and the Middle East [51]. Various countries across parts of Europe and Scandinavia have similar EMS systems, such as Sweden and the Netherlands [52,53,54]. Articles were included if they addressed elements of education or training related to paramedics in the setting of low-acuity clinical conditions and the use of non-conveyance decision support tools and/or alternative care pathways. Primary research studies utilising quantitative, qualitative, and mixed-method designs were included. Only peer-reviewed full-text articles published in English between 2002 and 2022 were considered. The search focussed on this timeframe as the literature reporting on lower acuity care and alternative pathways in EMS was increasing (18, 24–28). This period also represents a time of change for paramedicine, including registration as healthcare professionals, working in primary care settings, advances in clinical practice, and increasing educational requirements [1,55,56].

#### 2.1.2. Exclusion Criteria

Articles were excluded if they did not address elements of education or training provided to paramedics to support non-conveyance decision making and their use of low-acuity alternative care pathways. Staff working in ambulance services who did not meet the definition of a paramedic or equivalent were excluded. This included physicians, emergency call-takers, ambulance dispatchers, managers, and secondary triage clinicians. Armed forces’ paramedics/emergency medical technicians (EMT) were also excluded. 

#### 2.1.3. Protocol Amendment

The original scoping review protocol has been amended since publication to exclude the grey literature. The grey literature was excluded due to the variations and potential issues regarding the reproducibility of results presented by search engines such as Google Scholar [57,58]. Research by Gusenbauer and Haddaway [59] found that, after multiple queries, Google Scholar was not able to provide replicable results that could be explained by means of natural database growth. As a result, it was decided to exclude these sources. This also ensured that the search results were manageable within the appropriate timeframe of the review.

### 2.2. Information Sources

After a preliminary database search was completed and a consensus was reached regarding the keywords and search terms required to identify articles related to the topic, the search was conducted in the following databases: Scopus, CINAHL (EBSCO), Embase (Ovid), Emcare (Ovid), and MEDLINE (PubMed). 

### 2.3. Search Strategy 

The search was conducted on the 20 and 21 February 2023. The keywords and terms were divided into the following four categories:

Category one: paramedic OR paramedics OR “emergency medical technician” OR “emergency medical technicians” OR “ambulance officer” OR “ambulance officers” OR “ambulance clinician” OR “ambulance clinicians” OR “ambulance personnel” OR “ambulance staff” OR “ambulance nurse” OR “ambulance nurses” OR EMT.

Category two: education OR training OR “professional development” OR “staff development” OR “clinical education”.

Category three: ambulance OR “emergency medical service” OR “emergency medical services” OR prehospital OR “pre-hospital” OR “pre hospital” OR “out of hospital” OR “out-of-hospital” OR EMS OR “ambulance service”.

Category four: “low acuity” OR “low acuity patients” OR “non urgent” OR “non-urgent” OR “non conveyance” OR “non-conveyance” OR “ambulance transport” OR “non acute” OR “non-acute” OR “non transport” OR “non-transport” OR “discharged at scene” OR “medical necessity” OR “non-urgent patients” OR “non urgent patients”.

After searching each term separately and then combining them with ‘OR’ in each category, the main search string was created, which combined each category with the Boolean phrase ‘AND’. Appendix A, Table A1 provides the full search string for each database. The reference lists of the final included articles were also searched for any additional relevant literature.

### 2.4. Study Selection

The search results for each database were separately uploaded into Endnote™ 20 (Clarivate™, Philadelphia, PA, USA) and then imported into the Covidence™ systematic review software (Veritas Health Innovation, Melbourne, Australia. Available at www.covidence.org) [60]. The initial title and abstract screenings were independently conducted in Covidence™ by two authors (A.C. and D.G.E.), with a third author resolving any conflicts (A.M.W.). The same review process was employed for the full-text screenings. The selected articles were evaluated against the eligibility criteria. 

### 2.5. Data Charting

Article data were extracted into an Excel (Microsoft Corporation, Redmond, WA, USA) spreadsheet using a charting table developed by the research team. Data extraction and charting was conducted by one reviewer (A.C.) and verified by a second reviewer (D.G.E.) [46]. Any disagreements were resolved through discussion, or with a third reviewer (A.M.W.). The article data that were charted included author, country of origin, year of publication, article title, study aims, study design, setting and population, education method and approach, and findings.

### 2.6. Reporting and Synthesis of the Results

Descriptive statistics and a narrative summary of the included articles were used to characterise the research by country of origin, methodology, and study population. The results were grouped into three categories including non-conveyance outcomes, decision support tools and alternative care pathways, and education and training. 

The narrative synthesis discussed the main findings related to the research question. It was not the intention of this scoping review to conduct a meta-analysis or provide direct statistical comparisons, which may have been inappropriate due to the variations in study design, educational approach, and characteristics of the participants, including their differences in scope of practice. As the summary is descriptive, caution is advised when interpreting these findings, especially considering the heterogeneity of the included articles.

## 3. Results

### 3.1. Study Selection

**Figure 1:**
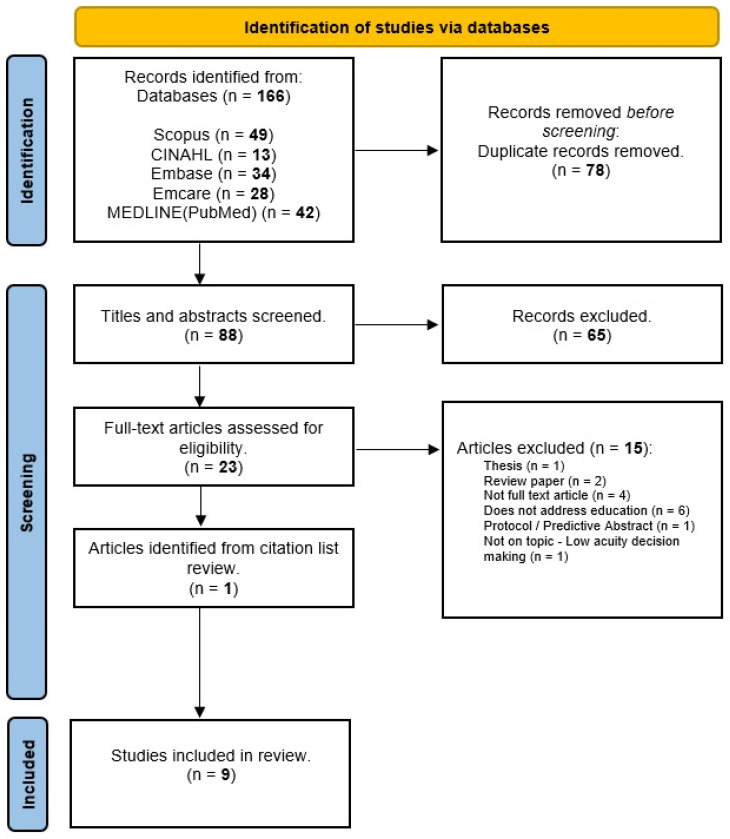
PRISMA Flow diagram.

The database search retrieved a total of 166 records. After removing 78 duplicates, title and abstract screening occurred for 88 records, with 65 excluded. Twenty-three records underwent full-text screening with fifteen excluded, leaving eight articles for inclusion. After reviewing the reference lists of these eight articles, one further study was identified. In total, nine articles were included in this scoping review (Figure 1).

### 3.2. Study Characteristics

Of the final nine included articles, seven were conducted in the UK [7,21,23,26,61,62,63], one in the USA [22], and one in Sweden [38]. Five articles employed qualitative methods [7,23,26,38,62], one mixed methods [61], and three were quantitative studies [21,22,63]. Two articles investigated the same treat-and-refer protocol implementation [21,26]. Seven articles reported on paramedics and emergency medical technicians. One article also included ambulance managers in their study population [7], and another focused on ambulance nurses [38]. A summary of the included articles is presented in Appendix B, Table A2. The study outcomes were grouped into the three categories listed below.

#### 3.2.1. Non-Conveyance Outcomes

Three quantitative articles [21,22,63] and one mixed-methods article [61] included the evaluation of changes in non-conveyance outcomes. Two of these, one of which was a pilot study [63], focused on paramedics who had undertaken extended care university education [61,63]. In the study by Cooper et al. [61], a small cohort of four paramedics were appointed to the role of extended care practitioner (ECP). They evaluated the effectiveness and benefit of the ECP role compared to a group of non-specialist paramedics. The authors reported that the ECPs treated 28% (n = 48, N = 170) of patients on-scene and reduced unnecessary conveyance to the ED compared to the paramedics, who treated 18% (n = 59, N = 331) of their patients at the scene. The ECPs conveyed 50% (n = 85, N = 170) of patients compared to the 64% (n = 212, N = 331) of patients for the paramedics. The ECPs attended to more low-acuity case types owing to their method of dispatch, including self-activation and ambulance crew referrals.

The research by Pilbery et al. [63] was conducted in a small sample of ten practicing specialist paramedics (SP). After attending a primary care placement, their non-conveyance rate increased by 35%. The SPs also attended to more appropriate low-acuity conditions suitable for their extended skillset.

Schaefer et al. [22] reported on a cohort of fire department basic life support EMTs [22] and their capability of identifying non-urgent cases suitable for an alternative destination. Compared to a matched control group, there was a decrease in patient conveyance to the ED (51.8% to 44.6%) and an increase in patients receiving care at a medical clinic (4.5% to 8.0%) or remaining at home (43.7% to 47.4%). 

Snooks et al. [21] investigated the implementation of 23 “treat-and-refer” protocols [21]. There was no significant difference in patient non-conveyance between the intervention and control groups when comparing patient age and clinical condition. The most common conditions attended to were falls, general illness or medical problems, and minor injuries.

#### 3.2.2. Decision Support Tools and Alternative Care Pathways

Five studies mentioned the use of various protocols or tools to guide non-conveyance assessment and decision making in low-acuity conditions. These included 23 “treat-and-refer” protocols [21], a clinical assessment tool (CAT) for falls [23], case-type eligibility criteria [22], non-conveyance guidelines [38], national guidelines, and a medical triage tool (Paramedic Pathfinder) [62]. It was found that some low-acuity decision support tools provided limited non-conveyance support and advice [38,62], or they were underused or not used to their full potential [21,23]. When non-conveyance was indicated, difficulties with referral processes, including limited access to alternative care pathways such as primary care, were frequently reported [7,23,26,38,62].

#### 3.2.3. Education and Training

Education and training approaches were reported in five articles [21,22,23,61,63]. Two articles described large-scale education programs that included the requirement for a university-based qualification for both ECPs and SPs with content specific to advanced patient assessment, extended care skills, health promotion, community health services, and a clinical placement in primary care [61,63]. Two articles addressed short in-service education programs [21,23]. One initially provided two days of training for 23 “treat-and-refer” protocols including protocol content, clinical roleplay, primary care services, referral, and research processes [21]. After this, two additional half-day workshops were required, addressing patient assessment, conveyance decision making, clinical scenarios, and a competency assessment [21]. The other article provided a two-hour training session on a falls’ clinical assessment tool (CAT), with content including falls’ aetiologies, older patient assessment, applying the tool, and research methods [23]. Lastly, one article mentioned training in alternative care destination eligibility criteria for minor illnesses and injuries including trauma, respiratory issues, musculoskeletal pain, and other conditions such as syncope and headache [22]. 

Of the other articles which referred to education, one was a follow-up study to the implementation of 23 “treat-and-refer” protocols, exploring the experiences of paramedics and EMTs whilst using them [26]. Another looked at paramedics’ experiences managing seizures, which included pre-existing guidelines and pathway tools to assist with transport decisions [62]. A further two articles reported on the non-conveyance experiences of ambulance nurses [38], and the other explored factors influencing care decisions made by paramedics [7]. Findings from these studies indicated a need for additional education and training in low-acuity conditions, assessment, and non-conveyance decision making [7,26,38,62].

## 4. Discussion

The aim of this scoping review was to explore the education and training provided to paramedics to support their use of alternative care pathways when managing lower-acuity clinical conditions. The education provided to paramedics found in this review ranged from an extended care university degree to short in-service training for various protocols and support tools. From the small number of articles identified, there appears to be limited research in this area of paramedic education. This is of interest considering that, over the past 20 years, calls to ambulance services for non-urgent illness and injury have been an increasing challenge in many EMS systems around the world. Over time, various approaches have been implemented to try and address this situation, including non-conveyance protocols and guidelines, secondary telephone triage services, and specialist community paramedic and practitioner roles. While these strategies have shown some success in managing a proportion of non-urgent patients, there has been limited evaluation of these strategies. 

### 4.1. Education and Training Approaches

There was considerable variation in the reported educational approaches used to support paramedics in assessing and managing low-acuity conditions. Education ranged from a two-hour training session up to an additional university-level qualification. In the two articles where ECPs and SPs had undertaken additional tertiary education which included advanced patient assessment and management, it was not surprising that they demonstrated higher non-conveyance rates [61,63]. It was remarked that graduate-level education and working in a minor injury unit had improved paramedics’ confidence and competence in clinical evaluations and decision-making [61]. The benefit of clinical placement time was also noted by Pilbery et al. [63], as it enabled SPs to enhance their knowledge of local healthcare pathways. In the articles by Snooks et al., paramedics also reported increased decision-making confidence, as well as improved patient assessment skills following in-service education on a suite of treat-and-refer protocols [21,26]. However, despite the introduction of these protocols and training, they found no significant difference in conveyance rates [21]. The paramedics in this study did not undertake clinical placement but did receive education from local primary care agencies. Two days of initial training were provided, but this proved to be inadequate. To address this, two half-day workshops were added, but the authors did not elaborate on exactly why this was required other than stating that it was “due to identified needs” [21]. While this extra training was clinically focused, it was still insufficient to influence the non-conveyance rates. This was reflected by the paramedics in the follow-up study by Snooks et al. [26], and, while the paramedics were positive about the protocols and the skills they had acquired, more in-depth training and in-field clinical support were necessary [26]. 

In the study by Halter et al. [23], paramedics underwent two hours of training time to use a falls’ assessment tool. In this qualitative study, the paramedics reported on their usage of the tool and its perceived value. However, in their post-implementation analysis, the tool was underutilised, and the paramedics reported that it provided little assistance with patient assessment and decision-making. This was interesting, considering the authors’ note that the paramedics in this study had not had previous training related to falls in older people or non-conveyance decision-making and were relying more on experience and intuition to guide transport decisions. As with the research by Snooks et al. [26], this suggests that short training options may not be sufficient to ensure sustained changes to practice, especially when there may be little perceived benefit to using decision support tools or non-conveyance is considered a high-risk activity. In the study by Schaefer et al. [22], the extent of EMT training to assess patients for ED avoidance was not well described. Prior to the active intervention phase of their study, there was a three-month period where the EMTs were to identify patients who could potentially be diverted to an alternative destination, but they were to transport to a hospital as per current practice. During this period, it was found that the EMTs were able to accurately identify a high percentage of patients eligible for alternative transport destinations, which was verified through physician reviews. During the intervention phase, the EMTs were then asked to offer their patients alternative transport options where appropriate. Subsequently, there was a modest decrease in ED conveyance and an increase in diversion to other care options. It is difficult to draw comparisons between the articles due to the heterogeneity of educational approaches as well as differences between international EMS systems, including paramedic accreditation requirements. However, it appears possible that lower-acuity patients can be appropriately diverted to alternative care pathways with suitable education.

### 4.2. Non-Conveyance Education and Training Needs

This review has highlighted the need for further education and training in non-conveyance assessment and decision-making. The articles that demonstrated higher non-conveyance rates were more associated with specialist paramedic roles requiring a significant volume of additional education. Shorter education approaches generally did not show positive outcomes in terms of conveyance rates or paramedic attitudes. This is supported in other research works that are not part of this review. Thompson [64] found that, despite differences in education, paramedics are adept in their assessment of emergency conditions but are less familiar and confident with the assessment skills required for low-acuity presentations. While certain aspects of assessment were considered universal to all cases, such as history-taking, other standard paramedic assessment approaches may be of limited value in lower-acuity patients. Despite patient assessment being pivotal to clinical decision-making, it was often inconsistent and unstructured, and, when medical conditions were not accommodated for in guidelines, patient assessment was often very limited [64]. When observing paramedics using a protocol which included a primary care physician referral pathway, Blodgett et al. [10] reported that some paramedics felt that they would be more confident to refer if they received training to improve their assessment skills. Johansson et al. [65] found that certain assessments, such as lung auscultation, blood glucose measurement, ECGs, and neurological and abdominal examinations, were often not performed by paramedics when they should be, even when they were indicated within a guideline. Some of the reasons cited for this included cultural practices, knowledge deficits, and cognitive biases from dispatch information and previous experiences, as well as low adherence to the “see-and-treat” guidelines and checklists. Consequently, additional education was implemented including feedback, lectures, and practical skills. 

The reviewed literature notes several challenges in low-acuity paramedic education. Whilst there is an obvious requirement to focus on the assessment and management of emergency conditions as part of the traditional paramedic role, further education to support paramedics in assessing and managing lower-acuity conditions is needed due to the increasing volume of these cases. Lederman et al. [38] identified this imbalance between emergency ambulance nurse education and the reality of clinical practice. It was remarked that, without the necessary non-conveyance education, nurses felt that their patient assessments may be prone to inaccuracies and errors. It was further commented, that for safe non-conveyance, a holistic patient assessment was crucial to avoid hasty decision making. However, due to the often-complex nature of non-conveyance situations, it was remarked that obtaining the entire picture of a patient would be ‘utopia’ and there may always be a piece of information missing despite their best efforts. Such educational gaps have been highlighted elsewhere, with the same disparities noted between higher volumes of training for emergency procedures compared to the training dedicated to lower-acuity presentations [6]. 

### 4.3. Education and Training Gaps

Apart from undertaking additional post-graduate university-based education, what has emerged from this review is that there are gaps in understanding the amount and type of education and training needed to support paramedics in assessing and managing low-acuity clinical conditions. The studies identified in this review that did provide in-service training for non-conveyance tools had several limitations that need to be considered when interpreting their results. This includes small sample sizes of paramedics and services, selection biases, convenience sampling, and short study time periods with no long-term follow-ups. In many cases, the information provided on education was limited, including no rationale for the chosen timeframe of training. For example, to use a falls’ assessment tool, paramedics were provided with two hours of training [23]. It is not clear if this timeframe was determined by the nature of the content, assumptions of paramedics’ decision making, operational demands, or other factors. In the study by Snooks et al. [21], it was also unclear how it was determined that two days of training would be sufficient. This was supplemented with additional clinically focused workshops, which the authors note were unanticipated. Despite the recognition by Snooks et al. [26] in their post-implementation research that more training was required and the noted complexities of changing practice, they made no recommendations as to how the training could be modified to address the challenges they found. It is apparent that prior to implementing alternative care pathway protocols or tools, training requirements may need to be tested and validated, including the content and duration with follow-up education, in-field support, and feedback provided to ensure sustained practice changes. 

It is worth noting that additional tertiary-based education consolidated through clinical placement does appear to positively influence non-conveyance decision making and improves patient assessment skills and confidence. However, while additional university-based education may be an appropriate requirement for paramedics to undertake extended care or practitioner roles, it is not necessary for all paramedics to be a specialist in community care, and not every low-acuity condition will require complex case management [3,66]. Whilst there are differences between and even within EMS systems and their education requirements across the world, there appear to be gaps in appropriate low acuity clinical education and training. It is vital that appropriate and practical training programs are developed and evaluated for the safe management of low-acuity conditions, which is consistent with the reality of clinical practice.

## 5. Limitations

There are several limitations in this scoping review. Despite using an array of search terms and sources to explore the peer-reviewed original research, only a small number of full-text published articles were eligible for inclusion. 

This review focused on the theme of paramedics’ education in relation to low-acuity conditions and alternative care pathways to avoid ED conveyance. However, several articles mentioned other challenges that can influence non-conveyance decision making, including organisational support, performance-based feedback mechanisms, scene time expectations, operational demand, and patient complexity [7,26,38,62]. Whilst exploring these elements was not the aim of this review, it is acknowledged that multiple factors contribute to non-conveyance decision-making; however, education is a major theme.

The intent of this review was to capture a range of articles over a 20-year time period. Any literature in this area prior to 2002 was excluded. However, the lower-acuity case load for ambulance services was less pronounced prior to this time and has been an increasing issue over the past 20 years. The reasons for this are multifactorial, including the lack of access to primary care and shortages of primary care physicians [67,68]. As a result, communities are relying more on ambulance services to fill the gap for their unmet healthcare needs, which is a contributor to the increasing demand for ambulance services worldwide [13].

Only studies published in the English language were included, which may have missed a wider international perspective from the literature related to low acuity care pathways published in other languages. Further, this may have also influenced the country of origin, as the nine included articles were from three countries in the northern hemisphere, with the majority of the studies being based in the UK.

## 6. Conclusions

The demand for ambulance services and paramedics to attend patients with lower-acuity clinical conditions has been increasing over the past two decades. This has resulted in the need for protocols and other tools to assist paramedics with low-acuity assessment, management, and conveyance decision-making. Despite the small number of articles eligible for this review, there was some limited evidence that appropriate and higher-level training not only improves ED non-conveyance but also increased paramedic competence and confidence in patient assessment and decision making. This is in contrast with short in-service training sessions, which in the majority of articles, did not demonstrate the same results. There was a notable gap between traditional emergency care training and what paramedics or ambulance clinicians face in actual clinical practice. This leads to the need to further explore what is a sustainable level of education and training required to develop patient assessment skills and decision-making confidence and support the use of low-acuity protocols and alternative care pathways, especially in the general emergency paramedic population.

## Figures and Tables

**Table 1:** PCC framework.

PCC Element	Definition
Population	Qualified paramedics as defined in the inclusion criteria.
Concept	Initial and ongoing education and training (if any) to inform the use of low acuity alternative care pathways where conveyance to the ED could be avoided, such as guidelines, protocols, triage, and/or referral tools.
Context	English language Anglo-American Emergency Medical Service (EMS) models Timeframe: 2002–2022

## Data Availability

Not applicable.

## Supplementary Materials

The following supporting information can be downloaded at: https://www.mdpi.com/article/10.3390/healthcare12020176/s1, Table S1: Preferred Reporting Items for Systematic reviews and Meta-Analyses extension for Scoping Reviews (PRISMA-ScR) Checklist.

## Appendix A

**Table A1:** Database search strings and records retrieved.

Database	Search Terms	Records Retrieved
Scopus	TITLE-ABS-KEY (paramedic) OR TITLE-ABS-KEY (paramedics) OR ((TITLE-ABS-KEY (“emergency medical technician”) OR TITLE-ABS-KEY (“emergency medical technicians”) OR ((TITLE-ABS-KEY (“ambulance officer”) OR TITLE-ABS-KEY (“ambulance officers”) OR ((TITLE-ABS-KEY (“ambulance clinician”) OR TITLE-ABS-KEY (“ambulance clinicians”) OR ((TITLE-ABS-KEY (“ambulance personnel”) OR ((TITLE-ABS-KEY (“ambulance staff”) OR ((TITLE-ABS-KEY (“ambulance nurse”) OR TITLE-ABS-KEY (“ambulance nurses”) OR ((TITLE-ABS-KEY (emt) AND (((TITLE-ABS-KEY (education) OR ((TITLE-ABS-KEY (training) OR ((TITLE-ABS-KEY (“professional development”) OR ((TITLE-ABS-KEY (“staff development”) OR ((TITLE-ABS-KEY (“clinical education”) AND (((TITLE-ABS-KEY (ambulance) OR ((TITLE-ABS-KEY (“emergency medical service”) OR TITLE-ABS-KEY (“emergency medical services”) OR ((TITLE-ABS-KEY (prehospital) OR TITLE-ABS-KEY (pre-hospital) OR TITLE-ABS-KEY (“pre hospital”) OR ((TITLE-ABS-KEY (“out of hospital”) OR TITLE-ABS-KEY (“out-of-hospital”) OR ((TITLE-ABS-KEY (ems) OR ((TITLE-ABS-KEY (“ambulance service”) AND (((TITLE-ABS-KEY (“low acuity”) OR ((TITLE-ABS-KEY (“low acuity patients”) OR ((TITLE-ABS-KEY (“non urgent”) OR TITLE-ABS-KEY (“non-urgent”) OR ((TITLE-ABS-KEY (“non conveyance”) OR TITLE-ABS-KEY (“non-conveyance”) OR ((TITLE-ABS-KEY (“ambulance transport”) OR ((TITLE-ABS-KEY (“non acute”) OR TITLE-ABS-KEY (“non-acute”) OR ((TITLE-ABS-KEY (“non transport”) OR TITLE-ABS-KEY (“non-transport”) OR ((TITLE-ABS-KEY (“discharged at scene”) OR ((TITLE-ABS-KEY (“medical necessity”) OR ((TITLE-ABS-KEY (“non urgent patients”) OR TITLE-ABS-KEY (“non-urgent patients”).	49
CINAHL (EBSCO)	“paramedic” OR TI “paramedic” OR AB “paramedic” OR “emergency medical technician” OR TI “emergency medical technician” OR AB “emergency medical technician” OR “emergency medical technicians” OR TI “emergency medical technicians” OR AB “emergency medical technicians” OR “ambulance officer” OR TI “ambulance officer” OR AB “ambulance officer”“ OR ambulance officers” OR TI “ambulance officers” OR AB “ambulance officers” OR “ambulance clinician” OR TI “ambulance clinician” OR AB “ambulance clinician” OR “ambulance clinicians” OR TI “ambulance clinicians” OR AB “ambulance clinicians” OR “ambulance personnel” OR TI “ambulance personnel” OR AB “ambulance personnel” OR “ambulance staff” OR TI “ambulance staff” OR AB “ambulance staff” OR “ambulance nurse” OR TI “ambulance nurse” OR AB “ambulance nurse” OR “ambulance nurses” OR TI “ambulance nurses” OR AB “ambulance nurses” OR “EMT” OR TI “EMT” OR AB “EMT” AND “education” OR TI “education” OR AB “education” OR “training” OR TI “training” OR AB “training” OR “professional development” OR TI “professional development” OR AB “professional development” OR “staff development” OR TI “staff development” OR AB “staff development” OR “clinical education” OR TI “clinical education” OR AB “clinical education” AND “ambulance” OR TI “ambulance” OR AB “ambulance” OR “emergency medical service” OR TI “emergency medical service” OR AB “emergency medical service” OR “emergency medical services” OR TI “emergency medical services” OR AB “emergency medical services” OR “prehospital” OR TI “prehospital” OR AB “prehospital” OR “pre-hospital” OR TI “pre-hospital” OR AB “pre-hospital” OR “pre hospital” OR TI “pre hospital” OR AB “pre hospital” OR “out of hospital” OR TI “out of hospital” OR AB “out of hospital” OR “out-of-hospital” OR TI “out-of-hospital” OR AB “out-of-hospital” OR “EMS” OR TI “EMS” OR AB “EMS” OR “ambulance service” OR TI “ambulance service” OR AB “ambulance service” AND “low acuity” OR TI “low acuity” OR AB “low acuity” OR “low acuity patients” OR TI “low acuity patients” OR AB “low acuity patients” OR “non-urgent” OR TI “non-urgent” OR AB “non-urgent” OR “non-conveyance” OR TI “non-conveyance” OR AB “non-conveyance” OR “non conveyance” OR TI “non conveyance” OR AB “non conveyance” OR “ambulance transport” OR TI “ambulance transport” OR AB “ambulance transport” OR “non acute” OR TI “non acute” OR AB “non acute” OR “non transport” OR TI “non transport” OR AB “non transport” OR discharged at scene” OR TI “discharged at scene” OR AB “discharged at scene” OR “medical necessity” OR TI “medical necessity” OR AB “medical necessity” OR “non-urgent patients” OR TI “non-urgent patients” OR AB “non-urgent patients”	13
Embase (Ovid)	paramedic*.ab. or paramedic*.ti. OR “emergency medical technician*”.ab. or “emergency medical technician*”.ti. OR “ambulance officer*”.ab. or “ambulance officer*”.ti. OR “ambulance clinician*”.ab. or “ambulance clinician*”.ti. OR “ambulance personnel”.ab. or “ambulance personnel”.ti. OR “ambulance staff”.ab. or “ambulance staff”.ti. OR “ambulance nurse*”.ab. or “ambulance nurse*”.ti. OR EMT.ab. or EMT.ti. AND education.ab. or education.ti. OR training.ab. or training.ti. OR “professional development”.ab. or “professional development”.ti. OR “staff development”.ab. or “staff development”.ti. OR “clinical education”.ab. or “clinical education”.ti. AND ambulance.ab. or ambulance.ti. OR “emergency medical service”.ab. or “emergency medical service”.ti. OR prehospital.ab. or prehospital.ti. OR “pre hospital”.ab. or “pre hospital”.ti. OR EMS.ab. or EMS.ti. OR “ambulance service”.ab. or “ambulance service”.ti. OR out-of-hospital.ab. or out-of-hopsital.ti. AND “low acuity”.ab. or “low acuity”.ti. OR “low acuity patients”.ab. or “low acuity patients”.ti. OR “non urgent”.ab. or “non urgent”.ti. OR “non conveyance”.ab. or “non conveyance”.ti. OR “ambulance transport”.ab. or “ambulance transport”.ti. OR “non acute”.ab. or “non acute”.ti. OR “non transport”.ab. or “non transport”.ti. OR “discharged at scene”.ab. or “discharged at scene”.ti. OR “medical necessity”.ab. or “medical necessity”.ti. OR “non urgent patients”.ab. or “non urgent patients”.ti.	34
Emcare (Ovid)	paramedic*.ab. or paramedic*.ti. OR “emergency medical technician*”.ab. or “emergency medical technician*”.ti. OR “ambulance officer*”.ab. or “ambulance officer*”.ti. OR “ambulance clinician*”.ab. or “ambulance clinician*”.ti. OR “ambulance personnel”.ab. or “ambulance personnel”.ti. OR “ambulance staff”.ab. or “ambulance staff”.ti. OR “ambulance nurse*”.ab. or “ambulance nurse*”.ti. OR EMT.ab. or EMT.ti. AND education.ab. or education.ti.OR training.ab. or training.ti. OR “professional development”.ab. or “professional development”.ti. OR “staff development”.ab. or “staff development”.ti. OR “clinical education”.ab. or “clinical education”.ti. AND ambulance.ab. or ambulance.ti. OR “emergency medical service”.ab. or “emergency medical service”.ti. OR prehospital.ab. or prehospital.ti. OR “pre hospital”.ab. or “pre hospital”.ti. OR EMS.ab. or EMS.ti. OR “ambulance service”.ab. or “ambulance service”.ti. OR out-of-hospital.ab. or out-of-hopsital.ti. AND “low acuity”.ab. or “low acuity”.ti. OR “low acuity patients”.ab. or “low acuity patients”.ti. OR “non urgent”.ab. or “non urgent”.ti. OR “non conveyance”.ab. or “non conveyance”.ti. OR “ambulance transport”.ab. or “ambulance transport”.ti. OR “non acute”.ab. or “non acute”.ti. OR “non transport”.ab. or “non transport”.ti. OR “discharged at scene”.ab. or “discharged at scene”.ti. OR “medical necessity”.ab. or “medical necessity”.ti. OR “non urgent patients”.ab. or “non urgent patients”.ti.	28
MEDLINE (PubMed)	(((((((((((((“paramedic”) OR (“paramedic”[Title/Abstract])) OR (“paramedics”)) OR (“paramedics”[Title/Abstract]) OR ((((“emergency medical technician”) OR (“emergency medical technician”[Title/Abstract])) OR (“emergency medical technicians”)) OR (“emergency medical technicians”[Title/Abstract]) OR ((((“ambulance officer”) OR (“ambulance officer”[Title/Abstract])) OR (“ambulance officers”)) OR (“ambulance officers”[Title/Abstract]) OR ((((“ambulance clinician”) OR (“ambulance clinician”[Title/Abstract])) OR (“ambulance clinicians”)) OR (“ambulance clinicians”[Title/Abstract]) OR ((“ambulance personnel”) OR (“ambulance personnel”[Title/Abstract]) OR ((“ambulance staff”) OR (“ambulance staff”[Title/Abstract]) OR ((((“ambulance nurse”) OR (“ambulance nurse”[Title/Abstract])) OR (“ambulance nurses”)) OR (“ambulance nurses”[Title/Abstract]) OR ((“EMT”) OR (“EMT”[Title/Abstract]) AND ((((((“education”) OR (“education”[Title/Abstract]) OR ((“training”) OR (“training”[Title/Abstract]) AND ((medline[Filter]) OR ((“professional development”) OR (“professional development”[Title/Abstract]) OR ((“staff development”) OR (“staff development”[Title/Abstract]) OR ((“clinical education”) OR (“clinical education”[Title/Abstract]) AND (((((((“ambulance”) OR (“ambulance”[Title/Abstract]) AND ((medline[Filter]) OR (((((“emergency medical service”)) OR (“emergency medical service”[Title/Abstract])) OR (“emergency medical services”)) OR (“emergency medical services”[Title/Abstract]) OR ((((((“prehospital”) OR (“prehospital”[Title/Abstract])) OR (“pre hospital”)) OR (“pre hospital”[Title/Abstract])) OR (“pre-hospital”)) OR (“pre-hospital”[Title/Abstract]) OR ((((“out of hospital”) OR (“out of hospital”[Title/Abstract])) OR (“out-of-hospital”)) OR (“out-of-hospital”[Title/Abstract]) OR ((“EMS”) OR (“EMS”[Title/Abstract]) OR ((“ambulance service”) AND (“ambulance service”[Title/Abstract]) AND (((((((((((((“low acuity”) OR (“low acuity”[Title/Abstract])) OR (“low-acuity”)) OR (“low-acuity”[Title/Abstract]) OR ((((“low acuity patients”) OR (“low acuity patients”[Title/Abstract])) OR (“low-acuity patients”)) OR (“low-acuity patients”[Title/Abstract]) OR ((((“non urgent”) OR (“non urgent”[Title/Abstract])) OR (“non-urgent”)) OR (“non-urgent”[Title/Abstract]) OR ((((“non conveyance”) OR (“non conveyance”[Title/Abstract])) OR (“non-conveyance”)) OR (“non-conveyance”[Title/Abstract]) AND ((medline[Filter]) OR ((“ambulance transport”) OR (“ambulance transport”[Title/Abstract]) OR ((((“non acute”) OR (“non acute”[Title/Abstract])) OR (“non-acute”)) OR (“non-acute”[Title/Abstract]) OR ((((“non transport”) OR (“non transport”[Title/Abstract])) OR (“non-transport”)) OR (“non-transport”[Title/Abstract]) OR ((“discharged at scene”) OR (“discharged at scene”[Title/Abstract]) OR ((“medical necessity”) OR (“medical necessity”[Title/Abstract]) OR ((((“non urgent patients”) OR (“non urgent patients”[Title/Abstract])) OR (“non-urgent patients”)) OR (“non-urgent patients”[Title/Abstract])	42

## Appendix B

**Table A2:** Summary of the included articles.

Authors, Publication Year, Country, and Title	Study Aims	Study Design	Setting and Population	Education Method/Approach	Findings
Cooper et al., 2004, UK [61]. The emerging role of the emergency care practitioner.	Evaluate the role, scope, impact, and benefit of emergency care practitioners compared to usual paramedic practice.	Qualitative and quantitative mixed-methods approach. Two stages of data collection: Stage 1—retrospective data analysis of reflective reports and patient documentation; Stage 2—individual and focus group interviews with ECPs, paramedics, ECP managers, and clinic staff.	South Western Ambulance Service NHS Trust (West Cornwall and West Devon). 15 ambulance staff, 4 Emergency Care practitioners, and 11 paramedics. ECPs worked half-time with an ambulance and half-time in a minor injury unit (MIU).	Two-year part-time Bachelor of Science (Emergency Care) including the following: seven modules including emergency care concepts and skills; and core paramedic practitioner competencies, e.g., advanced assessment and management. Upon completion of the degree, the graduates were appointed to a practitioner role.	Small sample size of participants and service locations. The ECPs managed 28% of patients on-scene compared to 18% by the paramedics. The ECPs conveyed 50% of patients compared to 64% by the paramedics. Reduced unnecessary trips by ambulance to ED. Different deployment methods. The ECPs were more likely to attend low-acuity cases compared to the paramedics through self-activation or referrals from ambulance crews. Case mix and referral process different between the ECPs and the paramedics. Additional clinical training improved practice, competence, confidence, leadership, and decision making with on-scene management, discharge, and referral. Working in MIU was essential to skill development and maintenance. Improved referral links with community-based primary and allied healthcare services. Evaluation of cost/benefit of extended scene times versus the cost of the conveyance required.
Halter et al., 2011, UK [23]. Complexity of the decision-making process of ambulance staff for assessment and referral of older people who have fallen: a qualitative study	Investigate ambulance staff decision-making experiences using the clinical assessment tool (CAT) flowchart to support conveyance decisions regarding older people who have fallen.	Qualitative. Semi-structured interviews.	London Ambulance Service NHS Trust. 12 ambulance staff interviewed after the study period: 1 paramedic, and 11 EMTs.	213 EMTs and paramedics trained across eight ambulance stations. Two-hour training session provided on using the CAT, the causes of falls, and older patient assessment.	Small sample size of participants, and EMTs were over-represented. Low levels of CAT usage. Generally not used to aid assessment and decision making, employed retrospectively after conveyance decisions had already been made. Experience, custom, and instinct was more influential on decision making. The participants reported difficulties with access to GPs. Access to other services, e.g., district nursing, was also problematic. Limited information on training. No follow up on the effectiveness of education and whether further training would be required to improve usage.
Lederman et al., 2019, Sweden [38]. Assessing non-conveyed patients in the ambulance service: a phenomenological interview study with Swedish ambulance clinicians.	Explore ambulance nurses’ experiences of assessing non-conveyed patients.	Qualitative. Open-ended interviews.	Stockholm County ambulance services. Eleven ambulance nurses: nine specialists and two registered.	Specialist ambulance nurses must have one year of additional university education.	Small participant sample size. Performing safe non-conveyance assessment was challenging, high-risk, with fear of making errors and causing harm. Need for improved access to medical records to facilitate holistic, safer, and more accurate patient assessment and decision making. Inadequate education and training in non-conveyance assessment and decision making. Non-conveyance not part of the ambulance curriculum. Ambulance education focus perceived as obsolete as it only prepares staff for acute conditions. Non-conveyance guidelines provide limited support, are unvalidated, and lack an evidence base. No performance-based feedback to guide practice improvement. Nurses prohibited from following up on patient outcomes. Performance cannot be improved without feedback. Difficulty arranging follow-up via primary care and home care. Health policy promotes alternate care pathways including non-conveyance. However, inadequate organisational support was observed. Experiences of feeling alone during non-conveyance assessments despite having access to a team including a colleague on scene and mandatory phone consult with a physician in the communications centre.
Noble et al., 2016, UK [63] Qualitative study of paramedics’ experiences of managing seizures: a national perspective from England.	Investigate the experiences of paramedics managing seizures including challenges, needs, and opinion on decision-making tools.	Qualitative. Semi-structured interviews with thematic analysis.	Five NHS Ambulance Trusts and education unit of professional body. 19 paramedics.	Additional education not part of the study methodology.	Small sample participant sample size. Most patients do not need conveyance to the ED. The paramedics felt that they were not adequately trained to manage seizures. Limited education on seizures in basic paramedic training. New higher education curriculum guidance developed but does not specify what should be covered or to what level. Low level of knowledge in caring for patients no longer seizing. National guidelines and pathfinder tool provide limited advice on post-seizure management and do not address on-scene challenges to care. Lack of alternative care pathways to avoid ED conveyance. GP and urgent care rarely available. Conveyance to ED often the only option. Perspectives of alternative healthcare providers and patients not explored. Pressure to minimise on-scene time influenced decision making in favour of conveyance to the ED. Lack of perceived organisational support if there is an adverse patient event from non-conveyance.
O’Hara et al., 2015, UK [7]. A qualitative study of systemic influences on paramedic decision making: care transitions and patient safety.	Explore the influences on paramedic decision making during patient care.	Qualitative multi-method two-phase qualitative study. Phase 1: document review and semi-structured interviews. Phase 2: On-road shift observation, digital diaries, and focus groups.	Three ambulance service trusts. Phase 1: 16 participants including ambulance directors, managers, and one front line specialist paramedic. Phase 2: shift observation of 33 paramedics, 7 specialist paramedics, and 18 EMTs/assistants.	Additional education not part of the study methodology.	Small sample size of paramedics and ambulance services. Organisational pressure to meet response times, reduce scene times, and decrease ED conveyance. Non-conveyance decisions more complex and time-consuming to assess and manage; conflicts with pressure to reduce scene and response times. Lack of awareness, availability, and access to alternative primary and community care pathways including ambulance community practitioners. Perspectives required from alternative care options, e.g., primary care to explore barriers to paramedic access. ED conveyance was the default option to avoid risks to paramedics (career) and patients (adverse events). Low confidence in organisational support if an adverse non-conveyance incident occurred. Training and education viewed as beneficial to decision making but impacted by operational demand and lack of organisational investment. Need for improved training and flexibility in scene times to ensure appropriate assessment and decision making. Work in isolation and limited access to formal decision-making support. Limited feedback on clinical decisions to improve performance.
Pilbery et al., 2022, UK [63]. The effect of a specialist paramedic primary care rotation on appropriate non-conveyance decisions (SPRAINED) study: a controlled interrupted time series analysis.	Determine if a primary care placement for specialist paramedics increased non-conveyance and was cost-effective.	Quantitative. Retrospective before and after comparison. Cost-effectiveness analysis.	Yorkshire Ambulance Service NHS Trust. 10 specialist paramedics with two years of experience in the role undertook a 10-week primary care placement.	Pre-existing university degree education in extended care with a short clinical placement time (30 days).	Single ambulance service and small sample size of specialist paramedics. Post-clinical placement increased appropriate non-conveyance compared to the control group. The specialist paramedics attended fewer cases post placement but attended to a higher proportion of lower-acuity case types due to dedicated tasking. Cost per SP non-conveyance decreased post GP placement. Working in primary care facilitated staff understanding of the healthcare system including alternative pathways. Difficult to generalise to other services as training varies between trusts, including the ad hoc development of specialist roles.
Schaefer et al., 2002, USA [22]. An emergency medical services program of alternate destination of patient care.	To determine if EMTs could decrease ED conveyance by appropriately identifying and triaging patients to alternative care destinations including physician-staffed urgent care centres and office-based practices.	Quantitative. Prospective cohort study compared with a historically matched retrospective control cohort.	Two fire department-based BLS agencies in King County EMS (Seattle). Two-phased approach. Phase 1: validated inclusion criteria and identified alternative referral pathways. Training provided to the EMTs. Phase 2: patients who met the criteria were offered alternative care pathways by the EMTs.	The EMTs trained in study goals, rationale, and patient case-type eligibility criteria. Unknown length of training.	Small sample size of EMS agencies. The paramedics were not trained in intervention. 5724 calls, 1016 (18%) met the intervention criteria. 453 patients (44.6%) conveyed to the ED, 482 (47.4%) not transported, 81 (8%) referred to and treated at an alternative destination. Out of the patients conveyed to the ED, 418 (n = 453) were eligible for alternative care. Decrease in ED conveyance post intervention (44.6% vs. 51.8%). Increase in alternative destination care, e.g., clinic or non-conveyance, i.e., home care. The patients who went to a medical clinic reported satisfactory care. EMTs can safely triage patients to alternative care options and reduce ED conveyance. Limited information on the education program. Did not state methods and duration of training, content, or who provided it. No follow up on the effectiveness of education and whether further training was required.
Snooks et al., 2004, UK [21] Towards primary care for non-serious 999 callers: results of a controlled study of ‘‘Treat and Refer’’ protocols for ambulance crews.	Develop and evaluate ‘‘treat-and-refer’’ (T&R) protocols allowing ambulance crews to leave patients at the scene with referral to community-based services or self-care advice.	Quantitative. Phase 1: T&R protocol development. Phase 2: Prospective cohort of T&R-trained ambulance crews (intervention group) from one station compared to crews using standard practice at another station (control group).	London Ambulance Service. Two ambulance stations in West London. Five paramedics and five EMTs trained to use protocols. 23 treat-and-refer protocols. Flowchart style accompanied by additional definitions and information.	Two-day training course. Facilitated by LAS trainers. Day 1: overview of protocols and clinical roleplay. Introduction to primary care services and referral processes. Day 2: Introduction and overview of primary care agencies and roles, including assessment and referral criteria. Need for accurate patient care records. Project context and research process. Two further half days of clinical training provided, led by the medical director and registrar. The clinical scenarios reviewed assessment and conveyance decision making. Emphasis on performing all protocol assessment criteria, systematic history, and patient observations. Each participant underwent a competency-based assessment.	Small sample size of participating ambulance stations. Crews were selected to participate based on their potential to be more compliant with the study. The study sites historically had high non-conveyance rates. Small cohort of patients during the intervention phase. Protocols used for 101 (N = 251) patients and 9 other patients who did not fulfil the inclusion criteria. No significant difference in non-conveyance rates compared to the control group. 17 out of 23 different protocols used. Falls most common, then soft tissue injury. A retrospective medical review found that 74 additional patients could have been included. 93 patients in the intervention group were conveyed to the ED. 36 had no ED investigations or received minor or no treatment. The crews used the protocols for 17 of these patients, no reason provided as to why they were conveyed. Increased scene time in the intervention group for both the conveyed (5 min) and non-conveyed patients (8 min). Improved clinical assessment documentation. Usage of protocols variable and not always used to their full potential. High patient satisfaction, particularly with the advice given.
Snooks, 2005, UK [26]. Gaps between policy, protocols and practice: a qualitative study of the views and practice of emergency ambulance staff concerning the care of patients with non-urgent needs.	Follow-up study from Snooks et al.’s 2004 study to investigate views on decision making pre- and post- implementation of treat-and-refer protocols to support non-conveyance and referral to alternative care.	Qualitative. Focus groups.	Two ambulance stations in West London. 21 paramedics.	As per Snooks et al.’s 2004 work.	Small paramedic sample size. The participants did not feel well-supported by the management and trainers. The crews felt that patient assessment was more systematic and reported increased confidence in their decision making. Additional effort and time needed to use the protocols. Practice more often driven by intuition and/or experience. Easier to convey patients to the ED. Referral processes to alternate pathways difficult. Had to persuade patients that referral to alternative pathway was more appropriate than going to the ED. Treat-and-refer protocols should be implemented throughout the whole ambulance system. Would make crews more systematic in their assessment and decision making. Training needs are underestimated and based on traditional ambulance practice, i.e., protocol-focussed, compared to education in other health professions that focusses on decision-making informed by clinical assessment and judgement. Primary care not a focus of traditional emergency ambulance training. Change management processes required to inform service delivery and implement new practices.
